# Sentinel node status in melanoma patients is not predicitive for overall survival upon multivariate analysis

**DOI:** 10.1038/sj.bjc.6602391

**Published:** 2005-02-08

**Authors:** F Roka, H Kittler, P Cauzig, C Hoeller, G Hinterhuber, K Wolff, H Pehamberger, E Diem

**Affiliations:** 1 Department of Dermatology, Division of General Dermatology, University of Vienna, Währinger Gürtel 18-20, Vienna A-1090, Austria

**Keywords:** melanoma, sentinel lymph node biopsy (SLNB), prognostic factors

## Abstract

Sentinel lymph node biopsy (SLNB) has become a widely accepted standard procedure in the staging of patients with cutaneous melanoma and absence of clinical lymph node metastases, although there is no final proof that SLNB influences overall survival in these patients. This study investigated the accuracy of SLNB and the clinical outcome of patients after a mean follow-up of 22 months. Between 1998 and 2003, SLNB was performed in 309 consecutive patients. Patients with one or more positive sentinel lymph nodes (SLNs) were subjected to selective lymphadenectomy (SL). Survival analyses were performed using the Kaplan–Meier approach. A Cox proportional-hazard analysis was used for univariate and multivariate analysis to explore the effect of variables on survival. Sentinel lymph nodes were identified in 299 of 309 patients (success rate: 96.8%). Of these, 69 (23%) had a positive SLN. The false-negative rate was 9.2%. Recurrence of disease to the regional lymph node basin (3.0%) and to the locoregional skin (2.6%) was rare in SLN-negative patients in contrast to SLN-positive patients (7.2 and 17.4%, respectively). The 3-year overall survival was 93 and 83% for SLN-negative and SLN-positive patients, respectively. Upon multivariate analysis, SLN status (*P*<0.001), Breslow thickness (*P*<0.02) and ulceration (*P*<0.026) were all found to be independent prognostic factors with respect to disease-free survival, whereas Breslow thickness proved to be the only significant factor with respect to overall survival.

Since its introduction by Morton in 1992 (Morton *et al*, 1992) sentinel lymph node biopsy (SLNB) has become a standard procedure in the staging and treatment of primary melanoma ⩾1 mm tumour thickness and clinically negative regional lymph nodes. According to Morton, the sentinel lymph node (SLN) can be defined as ‘the lymph node nearest the site of the primary melanoma, on the direct drainage pathway’.

Primary aims of this procedure were (i) to ascertain individual lymphatic drainage patterns of the primary tumour towards one or more different lymph node basins and (ii) to detect patients with micrometastatic lymphatic disease for selective lymphadenectomy (SL), which has been beneficial at least in subsets of melanoma patients (Balch *et al*, 1996).

Indeed SLN status has been found to be the strongest prognostic factor for survival and recurrence in patients with melanoma and clinically negative lymph nodes in a multicentre trial with 612 patients (Gershenwald *et al*, 1999). This observation was also taken into account at the revision of the staging system for cutaneous melanoma under the auspices of the American Joint Committee on Cancer (AJCC) in 2001. They concluded that ‘the information obtained from examining the sentinel node has an important impact on the staging of the disease, treatment planning, and the conduct of clinical trials in melanoma patients’ (Balch *et al*, 2001).

We have performed SLNB in more than 300 patients with melanoma stage I/II, all according to the same protocol. In this cohort of patients, we evaluated the accuracy of this procedure and the effect of SLN status on disease-free and overall survival.

## PATIENTS AND METHODS

Between 1998 and 2003, SLNB has been performed in 309 consecutive patients. All tumours were diagnosed by primary excision with a tumour margin ⩽0.5 cm. Indications for SLNB were Breslow tumour thickness ⩾1 mm, Clark level ⩾IV, ulceration and lesions with signs of regression or subungual localisation of the primary melanoma. Before SLNB, evidence of macrometastatic disease in regional lymph nodes or distant sites was ruled out by physical examination and individual staging procedures such as ultrasound, chest X-ray and computed tomography. Patients with tumour-positive SLNs were subjected to SL. All patients with primary melanoma ⩾1.5 mm thickness were considered for low-dose IFN*α*-2b therapy and patients with additional positive lymph nodes upon SL were considered for adjuvant high-dose interferon therapy according to Kirkwood *et al* (1996). Patient characteristics are shown in Table 1. The mean age of patients was 55.8 years (range 18–86 years); 58% were male. Histolopathology showed nodular (124 patients, 40%) or superficial spreading melanoma (105 patients, 34%) in the majority of patients. Ulceration of the primary lesion was present in 65 patients (21%). The mean tumour thickness (Breslow) was 2.7 mm (range: 0.25–30 mm). Of these, five patients presented with primary lesions ranging from 4 to 10 mm and one patient with a primary melanoma of 30 mm tumour thickness. Most patients had melanoma of Clark level IV (208 patients, 67%). Breslow tumour thickness and Clark level were not taken into account in six patients because of regression (four patients) or subungual localisation (two patients) of the primary melanoma.

Lymphoscintigraphy according to Berger and Cascinelli (Berger *et al*, 1997; Cascinelli *et al*, 2000) was performed the day before surgery by intradermal administration of technetium-99m-labelled nanocolloid at a dose of 30–60 MBq (Nanocoll®; Sorin Biomedica, Saluggia, Italy) around the biopsy site. Dynamic and static images were obtained. Under general anaesthesia methylene blue dye (0.3–1 ml) was injected intradermally around the excisional scar. Sentinel lymph nodes were identified intraoperatively by their blue colour and/or radioactivity detected with a hand-held gamma probe (C-Trak® Surgical Guidance System, Morgan Hill, CA, USA). All blue nodes and all nodes ⩾10% of the most radioactive or ‘hottest’ node were considered SLNs. Subsequently, the previous melanoma excisional biopsy scar was excised with 1 or 2 cm margin, depending on the thickness of melanoma invasion (⩽2 and >2 mm, respectively).

Sentinel nodes were fixed in 10% neutral-buffered formalin and embedded in paraffin. Serial 4 *μ*m thick sections (average: 10 levels) were analysed by conventional histologic staining (haematoxylin and eosin (H&E)) along with immunohistochemical staining using antisera directed against the S-100 protein and the melanoma antigen HMB-45. After SL, dissected lymph nodes were analysed by H&E and immunohistochemistry by nonserial sectioning.

Follow-up was performed every 3 months by physical examination. Ultrasound of lymph node basins and abdomen as well as chest X-ray were performed every 6 months. Computed tomography, magnetic resonance imaging and FDG-PET (fluorine-18-2-fluoro-2-deoxy-D-glucose positron emission tomography) were performed in patients with unclear results suggestive of metastatic disease.

Continuous data were compared with the *T*-test or with the Mann–Withney test, as appropriate. The *χ*^2^ test or Fisher's exact test were used for the comparison of proportions. Survival analyses were performed according to the life tables method and according to the method described by Kaplan and Meier. Comparison of the survival between groups was performed with the log-rank test. A Cox proportional-hazard analysis was used for univariate and multivariate analysis to explore the effect of variables on survival. The SPSS 10.0 software package (SPSS Inc., Chicago, IL, USA) was used for all statistical analyses. All given *P*-values are two-tailed and a *P*-value of <0.05 was regarded to indicate statistical significance.

## RESULTS

Sentinel lymph nodes were found in 299 of 309 patients (success rate: (100–10/3.09)=96.8%). In 10 patients, no sentinel node could be identified by either lymphoscintigraphy or blue dye. None of these patients was subjected to immediate lymph node dissection. Eight patients are still alive without any sign of continuing disease, one patient died of distant metastatic disease without evidence of lymph node metastasis and one patient was subjected to SL upon appearance of clinically positive lymph nodes after a follow-up of 20 months. Sentinel nodes were identified in one, two or three regional lymph node basins in 264 (85.4%), 30 (9.7%) and five patients (1.62%), respectively (Table 2). Among the five patients mapped in three different lymph node basins, four had primary melanoma in the lumbal region and one in the scapular region. Extra-anatomic SLNs were found in 10 of 299 patients (3.2%).

In 299 patients, a total of 640 (mean=2.1) SLNs were collected. In almost 60% of SLNB, two or more sentinel nodes were identified as either hot or blue (Table 2). Postoperative complications occurred in 7% and were mostly transient including seroma/haematoma or wound infection.

Patient characteristics stratified by SLN and SL status are shown in Table 3. Positive SLNs were identified in 69 patients (23%). Furthermore, seven patients developed recurrence in a lymph node basin that was negative by SLNB. This yields a false-negative SLNB rate of 9.2% (seven of (69+7)) and a failure rate of 2.2% (seven of 309). Ulceration, Clark level >II and Breslow thickness were all significant variables on SLN status. Probability of finding a positive SLN increased from 4% (one of 24 patients) in patients with tumour thickness ⩽1 mm up to 50% in patients with tumour thickness of greater than 4 mm (24 of 48 patients; *P*<0.001).

Of 69 patients with one or more positive SLN, 67 were subjected to SL (two patients refused further surgical treatment). Among the 67 patients with one or more metastatic SLN, 14 (21%) were found to bear further metastases in non-SLNs in the dissected basin upon SL. The median tumour thickness in these patients was 3.1 mm compared to 3.0 mm in patients with positive SLN only (NS, *P*=0.88) and 1.6 mm in SLN-negative patients (*P*<0.001).

Positive SLN specimens were histologically subdivided by bearing micro- (<2 mm in diameter) or macrometastastatic disease according to Carlson (Carlson *et al*, 2003; Table 4). Micrometastasis in the SLN was found in 47 (68%) and macrometastasis in 22 (32%) of 69 patients. In the group of micrometastatic positive SLN, seven (15%) out of 47 patients presented with further metastases in the same lymph node basin after SL, compared to seven (27%) of 22 patients with macroscopically positive SLNs (NS; *P*=0.26).

Of 309 patients, 49 (15.9%) developed disease recurrence during follow-up (Table 5). The median time to progression of disease was 18 months. In the group of patients with a positive SLN, 25 (36.2%) out of 69 patients developed recurrence: 12 (17.4%) to the locoregional skin, five (7.2%) to the draining lymph node basin and eight (11.6%) to systemic sites. In patients with a negative SLN finding, 22 (9.5%) developed recurrence: six (2.6%) patients to the locoregional skin, seven (3.0%) to the draining lymph node basin and nine (3.9%) to systemic sites.

**Figure 1:**
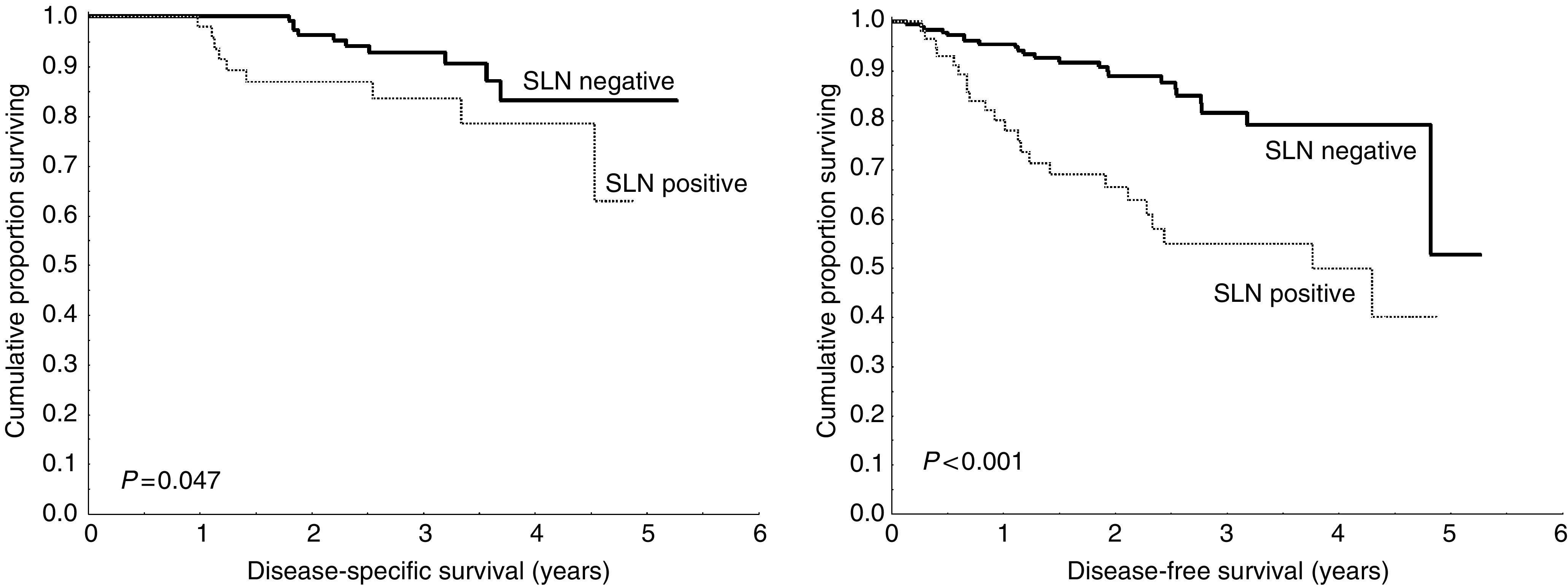
Kaplan–Meier curves for overall (left panel) and disease-free (right panel) survival for patients undergoing successful SLNB. The 3-year disease-free survival was 82% and 55% for negative and positive SLN patients, respectively. The 3-year overall survival was 93 and 83% for negative and positive SLN patients, respectively.

To date, 20 (6.5%) out of the 309 patients have died of metastatic disease. Of these, nine (3.9%, nine of 230) had a negative SLN, nine (13%, nine of 69) had a positive SLN and two (20%, two of 10) patients had an unknown sentinel node status. The 3-year overall and disease-free survival of the entire group of melanoma patients computed from the date of excision of the primary lesion were 90 and 75%, respectively. By univariate analysis, patients with a negative SLN had a significantly better disease-free (*P*<0.001) and overall survival (*P*<0.047) than patients with a positive SLN (Figure 1): The 3-year disease-free survival for negative and positive SLN patients was 82 and 55%, respectively. The 3-year overall survival for negative and positive SLN patients was 93 and 83%, respectively.

The model selected for multivariate regression was created by the forward stepwise elimination method. Analysis of several well-known prognostic factors with respect to disease-free survival are shown in Table 6: Positive SLN status (*P*<0.001), presence of ulceration (*P*=0.026) and Breslow tumour thickness (*P*=0.02) were all statistically significant prognostic factors by multivariate analysis, whereas Breslow thickness proved to be the only statistically significant prognostic factor with respect to overall survival (*P*=0.002).

## DISCUSSION

Since the first reports on SLNB for stage I and II melanoma lesions appeared, there has been an overwhelming enthusiasm for this technique. Several published series have shown various kinds of methods for identification, pathological examination and selection criteria for patients who could profit most from this staging method.

In our study, one or more SLNs were identified in 299 of 309 (96.8%) patients. As we did not perform synchronous SLNB and elective lymphadenectomy, false-negative rate of SLNB can only be estimated by recurrence of metastatic disease in the draining lymph node basin. Out of 209 patients with a negative SLN finding, seven patients (3.0%) developed a nodal recurrence in the previously mapped basin upon follow-up. One additional patient presented with metastases in the groin after being mapped in the ipsilateral axillary region. This yields a false-negative sentinel rate of 9.2% and a failure rate of 2.2%, which is in accordance with previous studies (Gershenwald *et al*, 1999; Morton *et al*, 1999; Morton *et al*, 2003; Vuylsteke *et al*, 2003). As previously shown by Gershenwald, detection failure of positive SLNs most commonly occurs because conventional histologic evaluation is unable to identify occult metastatic disease (Gershenwald *et al*, 1998). Furthermore, Morton reported a statistical lack in the detection of a positive SLN in patients with primary melanoma <2.01 mm: Compared to the incidence of regional lymph node metastasis in a historical control group of patients who where treated by wide local excision only (WLE), incidence of a positive SLN is only 60%. In contrast, this procedure is shown to detect accurately metastases from thicker lesions. New methods of sentinel node labelling, reflecting the microanatomy of the lymph node by the use of carbon dye, could decrease the false-negative rate of SLNB (Morton *et al*, 2003). The molecular analysis of the SLN using reverse transcription-polymerase chain reaction (RT–PCR) has been found to upstage a significant portion of SLN-negative patients. Since this method does not discriminate between melanoma metastases and nodal nevi, further investigations are needed until this procedure will become the standard of care.

Nevertheless, there is evidence that mechanisms other than failure of histopathologic examination may contribute to the failure of the SLNB technique in some patients. Li re-examined negative SLN specimens again and later of patients with regional recurrence and identified metastases in only seven of 12 false-negative SLNs in a group of 1152 patients (Li *et al*, 2003).

In our study, 20% of patients with positive SLN were found to have additional lymph node metastasis upon SL. Whether elective lymph node dissection is able to improve overall survival is still a matter of debate. In a single study, early detection of occult metastatic disease in the draining lymph node basin by elective (prophylactic) lymph node dissection has been shown to increase survival when compared to therapeutic (delayed) lymph node dissection (Cascinelli *et al*, 1998). On the other hand, Veronesi presented a prospective randomized clinical trial conducted by the WHO in which patients with melanoma of the limb did not benefit from prophylactic lymph node dissection (Veronesi *et al*, 1982). This study was further supported by Sim, who also observed no significant difference with respect to overall survival and metastasis-free survival when patients were split into three different groups treated with WLE alone, WLE and ‘immediate lymphadenectomy’, or WLE and ‘delayed lymphadenectomy’ (Sim *et al*, 1986). Nevertheless, 30% of patients with melanoma stage III survive more than 15 years after therapeutic lymphadenectomy (Balch *et al*, 2001), but this may not be addressed by surgical removal of lymph node metastases alone.

In our study, 15.9% of all patients developed recurrence of disease. Within a median follow-up of 22 months recurrence rate for SLN-positive patients (36.2%) was significantly higher compared to SLN-negative patients (9.5%). Several studies have addressed recurrence of disease for SLN-negative and -positive patients and showed comparable results, depending on the median follow-up period (Table 7).

Pattern of recurrence for SLN-negative patients reflects previous observations (Statius Muller *et al*, 2002), where relapse in the previously mapped lymph node basin is rare and equally as frequent as systemic metastasis. In contrast, systemic metastasis occurs in 11.6% of SLN-positive patients; however, the most frequent site of relapse in these patients was the locoregional skin (17.4%). Estourgie also found a substantially higher rate of local/in-transit metastases in SLN-positive patients compared to SLN-negative patients (23 and 7%, respectively). They suggested that this potentially inherent risk of the SLNB should be weighed against the possible survival benefit of early removal of nodal metastases (Estourgie *et al*, 2003). A possible explanation for high local recurrence rates in SLN-positive patients in our study may be excision margins of only 1 or 2 cm for primary melanoma below or above 1.5 mm in tumour thickness, respectively. As shown by Thomas, 1-cm margin of excision for melanoma with a poor prognosis (as defined by a tumour thickness of at least 2 mm) is associated with a significantly greater risk of locoregional recurrence than is a 3-cm margin. Nevertheless, overall survival was similar in the two groups (Thomas *et al*, 2004). Additionally, there might exist an inherent iatrogenic risk of SLNB and SL in SLN-positive patients: As described by Thomas and Clark (2004), patients having SLNB have approximately double the incidence of local/in-transit recurrence, while SLN-positive patients having SL have greater than four times the expected incidence.

In our series, 3-year disease-free and overall survival rates were 75.0 and 90.4%, respectively. Upon multivariate analysis, tumour thickness, ulceration and SLN status were all found to be significant factors with respect to disease-free survival. With respect to the overall survival upon multivariate analysis, tumour thickness was the only significant factor, and neither SLN status nor ulceration. This could be explained in part by the short follow-up period of our study. In contrast to this assumption, two other studies by Nowecki and Cascinelli with similar follow-up periods of 34 and 29 months, respectively, all showed a significant impact of SLN status on overall survival upon multivariate analysis (Cascinelli *et al*, 2000; Nowecki *et al*, 2003). Furthermore, Meier demonstrated that distant metastasis in patients with prior metastasis to lymph nodes already evolves after a median follow-up period of 25 months (Meier *et al*, 2002).

An alternative explanation for this finding could be that the patient characteristics in our study group differ from other studies as 3-year overall survival for SLN-positive patients was substantially higher in our study if compared to Gershenwald *et al* (1999) (83 *vs* 69.9%). This is not the case as the median tumour thickness of SLN-positive patients was identical in both studies (3.0 mm). Likewise, neither percentage of ulcerated tumours nor number of positive lymph nodes were substantially different from our study.

In conclusion, our study, including more than 300 patients, supports the influence of SLNB on disease-free survival, and is in accordance with previous studies in demonstrating the impact of tumour thickness, ulceration of the primary tumour and Clark level >2 on the occurrence of positive SLNs. In contrast to previous studies, no statistically significant correlation between SLN status and overall survival was observed in our study population.

## Figures and Tables

**Table 1:** Clinical and pathologic characteristics of the melanoma patient population (*n*=309)

	**No.**	**%**
*Sex*		
Female	131	42
Male	178	58

*Age*		
Mean	55.8	
Range	18–86	

*Site of primary tumour*		
Head and neck	15	5
Upper extremity	32	10
Lower extremity	74	24
Acral	24	8
Trunk	164	53

*Breslow thickness (mean: 2.7 mm; range: 0.25–30 mm)*		
⩽1 mm	41	13
1.01–2 mm	139	45
2.01–4 mm	80	26
>4 mm	43	14
Regr./subungual	6	2

*Clark level*		
II	8	3
III	70	23
IV	208	67
V	17	5
Regr./subungual	6	2

*Ulceration*		
Absent	241	78
Present	66	21
Subungual	2	1

*Histological subtype*		
Superficial spreading	105	34
Nodular	124	40
Acrolentiginous	18	6
Lentigous malignant	11	4
Desmoplastic	1	0.3
Unclassified	50	16

**Table 2:** Number of lymph node basins and number of SLNs at SLNB

	**Total**	**%**
*No. of basins*		
1	264	85
2	30	10
3	5	2
0	10	3

*No. of SLN*		
0	10	3
1	124	40
2	76	25
3	55	18
4	25	8
5	9	3
6	9	3

Total	309	100

SLN=sentinel lymph node; SLNB=sentinel lymph node biopsy.

**Table 3:** Patient characteristics stratified by SLN status and SL status

	**All patients**		**SLN negative**		**SLN positive**		**SLN and SL positive**	
	**No. of patients**	**%**	**No. of patients**	**%**	**No. of patients**	**%**	**No. of patients**	**%**
Total	299	100	230	77	69	23	14	20
*Sex*								
Male	171	57	126	74	45	26	10	22
Female	128	43	104	81	24	19	4	17

*Site of primary tumour*								
Trunk	158	53	120	76	38	24	7	18

*Clark level*								
II	8	3	8	100	0	0	0	0
III	71	24	59	83	12	17	1	8
IV	202	67	151	75	51	25	13	25
V	12	4	6	50	6	50	0	0
Regr./subungual	6	2	6	100	0	0	0	0

*Ulceration*								
Absent	226	76	180	80	46	20	9	20
Present	67	22	44	66	23	34	5	22
Regr./subungual	6	2	6	100	0	0	0	0

*Tumour thickness (mm)*								
⩽1	24	8	23	96	1	4	0	0
1.01–2	129	43	109	84	20	16	2	10
2.01–4	92	31	68	74	24	26	9	38
>4	48	16	24	50	24	50	3	13
Regr./subungual	6	2	6	100	0	0	0	0

SLN=sentinel lymph node; SL=selective lymphadenectomy.

**Table 4:** Distribution of patients according to SL status and positive SLN status

	**SLN-positive patients (*n*=69)**			
**SL**	**Micrometastatic**		**Macrometastatic**	
Negative	38	81%	15	73%
Positive	7	15%	7	27%
Not done	2	4%	0	0%

SLN=sentinel lymph node; SL=selective lymphadenectomy.

**Table 5:** First site of recurrence and death of disease

	**Locoregional skin**	**Draining LN basin**	**Systemic**	**Total recurrence**	**Death of disease**
SLN neg (*n*=230)	6 (2.6%)	7 (3.0%)	9 (3.9%)	22 (9.5%)	9 (3.9%)
SLN pos (*n*=69)	12 (17.4%)	5 (7.2%)	8 (11.6%)	25 (36.2%)	9 (13.0%)
SLN unknown (*n*=10)	0 (0%)	1 (10.0%)	1 (10.0%)	2 (20.0%)	2 (20.0%)

Total	18 (5.8%)	13 (4.2%)	18 (5.8%)	49 (15.8%)	20 (6.5%)

SLN=sentinel lymph node; LN=lymph node.

**Table 6:** Multivariate analysis of prognostic variables influencing disease-free and overall survival

	**Disease-free survival**			**Overall survival**		
	***P***	**HR**	**95% CI**	***P***	**HR**	**95% CI**
Tumour thickness	<0.001	1.067	1.010–1.127	0.002	1.098	1.036–1.165
Ulceration	0.003	2.230	1.103–4.509			
SLN status	<0.001	4.264	2.216–8.205			

HR=hazard ratio; CI=confidence interval.

**Table 7:** Report of recurrence rates in SLN-negative and -positive patients in the literature

**Author**	**SLN neg. (%)**	**SLN pos. (%)**	**Follow-up period (months)**
Fincher *et al* (2003)	10	26.3	50/38
Wagner *et al* (2003)	12.1	36.5	31.4
Chao *et al* (2002)	6.0	15.5	16
Gershenwald *et al* (1998), (1999)	11	—	35
Vuylsteke *et al* (2003)	14	55	72
Statius-Muller *et al* (2002)	11	46	38
Current study	9.5	36.2	22

SLN=sentinel lymph node.
